# LESSONS LEARNED FROM AN ENHANCED SERVICE COORDINATION MODEL IN SENIOR HOUSING

**DOI:** 10.1093/geroni/igac059.291

**Published:** 2022-12-20

**Authors:** Alexandra Hennessa, Robyn Stone

**Affiliations:** LeadingAge, Washington, District of Columbia, United States; LeadingAge, Washington, District of Columbia, United States

## Abstract

Residents of affordable housing communities are growing older, and new residents are moving in at older ages. As these residents age, their need for services and supports increase. Approximately 5,200 housing communities subsidized by the U.S. Department of Housing and Urban Development (HUD) have a service coordinator on-site to help connect residents with needed services and resources. In 2021, the LeadingAge LTSS Center @UMass Boston (LTSS Center) conducted a process evaluation of the LSA Senior Connect Model, which was designed to build the capacity of service coordinators to better meet the needs of aging residents. The study activities included a review of program-related documents, analysis of resident assessments, interviews with staff, resident focus groups, and cost analysis. We will summarize the model’s framework and tools, and discuss lessons learned from the process evaluation. Lessons for policy and practice will also be discussed.

